# Metabolic Characteristics and Diagnostic Contribution of ^18^F-FDG PET/CT in Gastric Carcinomas

**DOI:** 10.4274/mirt.galenos.2020.75537

**Published:** 2020-02-17

**Authors:** Esra Arslan, Tamer Aksoy, Cihan Gündoğan, Çiğdem Şen, Selda Yılmaz Tatar, Nevra Dursun, Tevfik Fikret Çermik

**Affiliations:** 1 University of Health and Sciences, İstanbul Training and Research Hospital, Clinic of Nuclear Medicine, İstanbul, Turkey; 2 Yeniyüzyıl University, Gaziosmanpaşa Hospital, Department of Nuclear Medicine, İstanbul, Turkey; 3 University of Health and Sciences, İstanbul Training and Research Hospital, Department of Pathology, İstanbul, Turkey

**Keywords:** Gastric cancer, 18F-fluorodeoxyglucose positron emission tomography/computed tomography (18F-FDG PET/CT), adenocarcinomas

## Abstract

**Objectives:**

The aim of this study was to evaluate ^18^F-fluoro-2-deoxy-glucose (FDG) uptake patterns in primary tumors and metastatic lesions, and also to assess the diagnostic contribution of positron emission tomography/computed tomography (PET/CT) in the initial staging of gastric cancer (GC).

**Methods:**

The total number of 341 patients with GC were included in this study. All ^18^F-FDG PET/CT imagings were performed for initial staging. The maximum standardized uptake value (SUV_max_) of primary tumor, obtained from ^18^F-FDG PET/CT imaging was compared between subtypes of GC.

**Results:**

Mean SUV_max_ of 339 patients’ primary tumor was 12.9±8.6. The highest mean SUV_max_ was detected in patients with medullary subtype GC (17.8±9.9) while the lowest mean SUV_max_ (9.7±7.6) was seen in signet ring cell carcinoma (SRCC). The primary mean SUV_max_ was found statistically higher in tubular adenocarcinoma (TAC) group than SRCC group (p<0.001). Higher SUV_max_ values were found statistically significantly correlated with advanced age (aged ≥60) and increased tumor size (>3 cm) in patients with TAC (p=0.03). Primary tumor SUV_max_ was found statistically higher in regional lymph node (RLN) positive patients than in RLN negative patients in TAC and SRCC groups (p<0.001 and p=0.012, respectivelly). Also, in patients with SRCC, SUV_max_ was significantly higher in the distant metastatic group than in the group without metastasis (p=0.025).

**Conclusion:**

Increased primary tumor SUV_max_ was associated with some of clinical parameters such as age and RLN metastasis in patients with TAC. However, there was no relationship between distant metastatic state and primary tumor ^18^F-FDG uptake in TAC. However, high SUV_max_ of primary tumor in SRCC was associated with regional and distant metastasis, and primary tumor ^18^F-FDG uptake may be a prognostic value for this subgroup.

## Abstract (tr)

**Amaç:**

Bu çalışmanın amacı primer tümörlerde ve metastatik lezyonlarda ^18^F-floro-2-deoksi-glukoz (^18^F-FDG) tutulum paternlerini değerlendirmek ve ayrıca mide kanserinin (MK) evrelemesinde pozitron emisyon tomografi/bilgisayarlı tomografi’nin (PET/BT) tanısal katkısını değerlendirmektir.

**Yöntem:**

Çalışmaya toplam 341 MK hastası dahil edildi. Primer evreleme ^18^F-FDG PET/BT görüntüleme ile yapıldı. ^18^F-FDG PET/BT görüntülemeden elde edilen primer tümöre ait maksimum standart tutulum (SUV_max_) MK alt tipleri arasında karşılaştırıldı.

**Bulgular:**

Üç yüz otuz dokuz hastaya ait primer tümörün ortalama SUV_max_ değeri 12,9±8,6 idi. En yüksek ortalama SUV_max_, medüller alt tip MK’li hastalarda (17,8±9,9), en düşük ortalama SUV_max_ (9,7±7,6), taşlı yüzük hücreli mide kanserinde (TYHMK) görüldü. Ortalama SUV_max_, tubuler adenokarsinom (TAK) grubunda TYHMK grubundan istatistiksel olarak daha yüksek bulundu (p<0,001). Daha yüksek SUV_max_ değerleri, TAK’li hastalarda ileri yaşla (yaş ≥60) ve artan tümör büyüklüğü (>3 cm) ile istatistiksel olarak anlamlı derecede ilişkili bulundu (p=0,03). Primer tümör SUV_max_, bölgesel lenf nodu (RLN) pozitif olan hastalarda TAK ve TYHMK gruplarındaki RLN negatiflerden anlamlı olarak yüksek bulundu (p<0,001, p=0,012, sırasıyla). Ayrıca, TYHMK’lı hastalarda, uzak metastatik grupta SUV_max_, metastazı olmayan gruba göre anlamlı derecede yüksekti (p=0,025).

**Sonuç:**

Primer tümör SUV_max_ı yüksek TAK’li hastalarda yaş ve RLN metastazı gibi bazı klinik parametrelerle ilişkilendirildi. Bununla birlikte, TAK’de uzak metastatik durum ile primer tümör ^18^F-FDG tutulumu arasında ilişki bulunmadı. Bununla birlikte, TYHMK’deki primer tümörün yüksek SUV_max_’ı, bölgesel ve uzak metastaz ile ilişkiliydi ve primer tümörün ^18^F-FDG tutulumu, bu alt grup için prognostik bir değeri olabileceğini düşünmekteyiz.

## Introduction

Gastric cancer (GC) is the fifth most common cancer worldwide with an estimated 900.000 new cases diagnosed annually (1). Tubular adenocarcinomas (TAC), the most prevalent GC subtype, is the third leading cause of cancer-related deaths (2). The main issue pointed out by reports is that GC typically constitutes higher proportion of new mortality/cases compared with more prevalent cancers (3). The majority of patients with GC (64%) are usually diagnosed when the disease is already in advanced or metastatic stages (4).

Recently, ^18^F-fluoro-2-deoxy-glucose positron emission tomography/computed tomography (^18^F-FDG PET/CT) has been demonstrated as a noninvasive, useful modality for diagnosis and staging of patients with cancer (5). The higher maximum standardized uptake value (SUV_max_) levels were found significantly associated with the metastasis and poor prognosis in several types of cancer, including breast, esophagus and non-small cell lung cancers (6,7,8).

The role of ^18^F-FDG PET/CT in GC remains controversial, as reports indicate low sensitivity for staging and predicting prognosis (5). In contrast to limited sensitivity reports, several studies concluded an acceptable prognostic and clinical value of ^18^F-FDG PET/CT in GC staging (9,10).

In this study, we aimed to evaluate ^18^F-FDG uptake patterns in GC subtypes, not only in primary tumors but also in nodal and distant metastatic lesions, as well as to assess the diagnostic contribution of PET/CT to nodal involvement and distant metastasis in the initial staging of GC.

## Materials and Methods

### Patients

The total of 341 patients with GC [256 (75.1%) males, 85 (24.9%) females, mean age 62.2±11.5 years (range: 23-90)], who were diagnosed as having primary GC with gastroscopy, histopathological examination and underwent ^18^F-FDG PET/CT for initial staging between May 2011 and July 2018 were included in this study. Patients who were previously diagnosed as having another malignancy were not included in the study.

Primary GC diagnosis and histopathological analysis have been based on tissue samples derived by endoscopic biopsies performed before ^18^F-FDG PET/CT imaging. ^18^F-FDG PET/CT imagings were performed preoperatively or before chemotherapy/radiotherapy for all patients. Staging was performed based on the TNM classification for carcinoma of the stomach according to the 8^th^ edition of the American Joint Committee on Cancer guidelines (11). The staging system depends on extend of the tumor, regional lymph node (RLN) and distant metastasis. Also, other prognostic factors such as tumor diameter, histological grade, lymphovascular invasion, perineural invasion, surgical margins were evaluated pathologically on resection specimens. The histological classification proposed by the Word Health Organization was used for pathological reporting (12). This retrospective study was approved by the local ethics comittee (2017/1048). All patients included were asked for their verbal or written consent for the use of their individual clinical findings for research purposes.

### ^18^F-FDG PET/CT Imaging

Patients with blood glucose levels lower than 150 mg/dL after at least six hours of fasting were admitted for the procedure. Standard 3.7-5.2 MBq/kg (0.1-0.2 mCi/kg) ^18^F-FDG intravenous injection was administered to the patients. Sixty minutes after 4 injection of ^18^F-FDG, whole body PET/CT imaging was obtained including the area from vertex to upper femur at supine position (first 42 imagings were performed by Biograph 6 HD LSO, and subsequent 299 imagings were performed by mCT 20 ultra HD LSO PET/CT), (Siemens molecular imaging, Hoffmann Estates, Illinois, USA). A solution containing 75 cc mannitol and 2 grams of locust bean gum was added to 1.5 liters of water for all patients to drink as negative oral contrast agent during the time period between injection and image acquisition. CT imaging for PET/CT was performed using a multi-detector scanner with 6 and 20 slices, at 80-140 kV, 20-266 mAs, 0.8 pitch and 512x512 matrix [personalized settings determined by automatic exposure control system; automatically defined by the software used by manufacturer (CareDose 4D) depending on the patient and region assessed]. CT imaging was performed between vertex and upper-thigh in craniocaudal direction with 5 mm of slice thickness and 0.5 seconds of rotation time. Then, PET imaging was performed in the same range through craniocaudal direction at 8 to 9 bed positions, 1.5 minutes for each PET bed using Siemens mCT 20 ultra HD LSO PET-CT scanner. Ultra HD images were acquired using Time of flight + True X algorithm for Siemens mCT 20 ultra HD LSO PET-CT at iteration 2 and subset 16 values for reconstruction. 3D imaging was performed using Siemens Biograph 6 HD LSO PET-CT scanner at 6 to 8 bed positions for 2.5 minutes per bed. HD images were acquired using True X algorithm for Siemens Biograph 6 HD LSO PET-CT.

### Interpretation of PET/CT Images

Images acquired from all patients were evaluated by at least two senior nuclear medicine physicians, at the workstation both visually and semi-quantitatively in axial, coronal and sagittal planes. ^18^F-FDG PET/CT image evaluation was done unaware of previous imaging results of subjects. For visual evaluation, foci of increased ^18^F-FDG uptake compared to background and CT findings were evaluated in conjunction. For semi-quantitative analysis, SUV_max_ was measured by placing the “volume-of-interest” around the ^18^F-FDG positive primary and nodal metastatic lesions in visual evaluation. Focal FDG uptakes with an abnormal soft tissue mass or a lymph node on CT counterpart was considered significant for malignancy. For SUV_max_ calculation, “regions of interest” (ROI) which included the location of highest uptake was drawn on PET cross-sections. SUV_max_ was calculated according to the following formula: Maximum activity inside the ROI (MBq/gr) /injected ^18^F-FDG dosage (MBq/kg body mass). Maximum tumor diameter and wall thickness were measured from the axial CT scan of the PET/CT imaging.

### Statistical Analysis

All the data were analyzed with SPSS software for Windows (v21.0; IBM, Armonk, NY, USA). Individual and aggregate data were summarized using descriptive statistics including mean, standart deviations, medians (minimum-maximum), frequency distributions and percentages. Normality of data distribution was verified by Kolmogorov-Smirnov test. Comparison of the variables with normal distribution was made with Student t-test. Evaluation of categorical variables was performed by chi-square test. The kappa statistic was calculated to evaluate the agreement. P values of <0.05 were considered statistically significant.

## Results

In our study group, the prevalence was highest in the patients’ seventh decade of life (37.2%), followed by the sixth decade (25.9%). In PET/CT imaging, 22.0% (n=75) of the lesions were detected in the proximal part (cardioesophageal junction or cardia), 29.6% (n=101) in the middle part (fundus and corpus), 38.7% (n=132) in the distal part (antral or pyloric) and 9.7% (n=33) of the lesions were diffuse in the stomach.

The final histopathologic diagnosis was obtained in 70.0% of patients (n=239) only by endoscopic biopsy. These patients were directed to non-surgical treatments due to inoperability. In this subgroup, the findings obtained by the second PET/CT were used as the gold standard in the following three or six months after diagnosis. Remaining 102 patients underwent gastrectomy and nodal staging was performed together with detailed histopathological analysis in these patients. TAC was the most common histological subtype, accounting for 62.7% (n=214) of total patients, followed by signet ring cell carcinoma (SRCC) (26.9%) (n=92), mucinous carcinoma (5.6%) (n=19), neuroendocrine carcinoma (1.5%) (n=5), adenosquamous carcinoma (0.9%) (n=3), medullary carcinoma (0.9%) (n=3) and other subtypes (1.5%) (n=5) in our study (Table 1). Histological subtypes of patients who underwent surgical resection were as follows: TAC in 57 (55.9%) patients, SRCC in 21 (20.6%) patients, mucinous carcinoma in 17 (16.6%) patients, neuroendocrine carcinoma in 4 (3.9%) patients, medullary carcinoma in 2 (2%) patients and adenosquamous carcinoma 1 (1%) patient.

**Figure 1:**
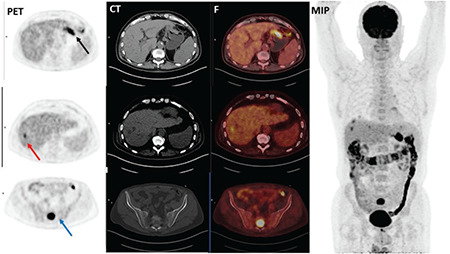
A 68-year old male patient with gastric tubular adenocarcinoma. Axial PET (A), CT (B), and fusion (C) images showed high ^18^F-FDG uptake (SUV_max_: 13.29) in primary tumor in the fundus of the stomach (black arrow). Liver metastasis showed increased ^18^F-FDG uptake (short axis diameter: 1.88 cm, SUV_max_: 6.24) (red arrow). Additionally, bone metastasis was demonstrated in PET/CT images (SUV_max_:16.29) (blue arrow) ^18^F-FDG: Fluorine-18-fluorodeoxyglucose, SUV_max_: Maximum standardized uptake value, MIP: Maximum intensity projection image, PET: Positron emission tomography, CT: Computed tomography

**Figure 2:**
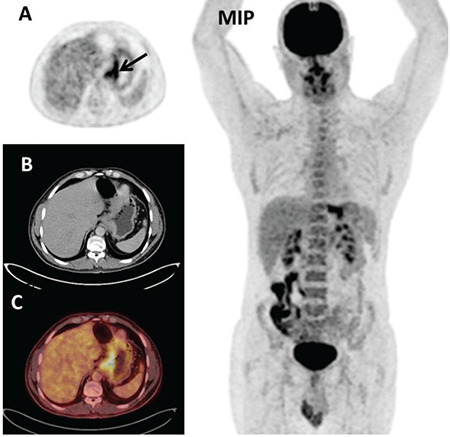
A 48-year-old male patient with SRCC. Axial PET (A), CT (B), and fusion (C) images showed ^18^F-FDG uptake (SUV_max_: 7.9) in primary tumor in the cardia of the stomach (arrow). There was no locoregional lymph node or distant metastasis in PET/CT imaging ^18^F-FDG: Fluorine-18-fluorodeoxyglucose, SUV_max_: Maximum standardized uptake value, MIP: Maximum intensity projection image, PET/CT: Positron emission tomography/computed tomography, SRCC: Signet ring cell carcinoma

Primary tumor FDG uptake was observed in all the subjects except 2 patients with SRCC. Therefore, the analysis was performed according to semiquantitative analysis instead of visual evaluation. Mean ± standard deviation SUV_max_ obtained from 339 patients with ^18^F-FDG accumulation in primary tumor was 12.9±8.6 in PET/CT imaging. The highest SUV_max_ was detected in patients with medullary subtype GC (17.8±9.9) while the lowest SUV_max_ (9.7±7.6) was seen in SRCC. A statistically significant difference was documented among all histological types based on ^18^F-FDG uptakes (p<0.001), and the primary tumor SUV_max_ was found statistically higher in patients with TAC (14.5±8.8) than in patients with SRCC (p<0.001) (Table 1) (Figure 1 and 2).

The SUV_max_ measured in group aged 60 years or over (n=147) was found to be statistically higher than in group aged lower than 60 years (n=67) in patients with TAC (p=0.03). When the primary tumor size was taken into consideration, the SUV_max_ of RLN positive group in PET/CT (n=168) was found significantly higher than RLN negative group (n=46) (15.9±8.8 and 8.7±5.9, respectively) (p<0.001). There were no statistically significant differences in terms of SUV_max_ among the different anatomic locations of the lesions in stomach (p=0.274), and different tumor differentiation grades in patients with TAC (p=0.102) (Table 2).

The primary tumor SUV_max_ of RLN positive group (n=62) was found significantly higher than RLN negative group (n=30) (11.0±8.5 and 6.9±3.8, respectively) in patients with SRCC (p=0.012). Similarly, the primary tumor SUV_max_ of the group with distant organ metastasis (n=11) was significantly higher than the group without distant organ metastasis (n=81) (14.1±8.2 and 9.7±7.3, respectively) in patients with SRCC (p=0.025). In patients with SRCC, there were no statistically significant differences in terms of primary tumor SUV_max_ among the different anatomic locations (p=0.284), and different tumor differentiation grades (p=0.946) (Table 3). In SRCC group, primary tumor FDG uptake was increased in the presence of distant nodal and distant organ metastasis. There was a similar tendency for distant nodal metastasis in the TAC group, but this was not true for distant organ metastasis in our study group (Table 2).

In our study, 102 patients underwent surgical resection. Postoperative histopathological analysis was accepted as gold standard for detection of RLN metastatic involvement and sensitivity and specificity for PET/CT were calculated according to postoperative histopathological analysis results. The sensitivity and specificity of PET/CT were found to be 78.2% and 58.3% in the detection of RLN, respectively. Positive predictive value (PPV) and net present value (NPV) of the PET/CT imaging were 89.5% and 45.2% for RLN metastasis, respectively. On the other hand, primary tumors’ SUV_max_ was found statistically higher in patients with positive RLN (14.6±8.9) than in patients with negative RLN (8.2±5.3) (p<0.001). The SUV_max_ of RLN was found significantly higher in patients with TAC than in patients with SRCC (SUV_max_=8.8±8.4 and 5.8±7.1, respectively; p=0.001) (Table 4).

Distant organ metastasis was found in 91 (26.7%) patients. Fourty two patients with distant metastasis had TAC, 11 had SRCC and 38 remaining patients had other subtypes of GC. In our study group, the most common organ with metastasis was found as liver (64.8%, n=59). This was followed by bone-bone marrow (11%, n=10), multiple organs (9.9%, n=9), lungs (8%, n=7) and serosal metastasis (6.3%, n=6). There was no relation between distant organ metastatic state and primary tumor ^18^F-FDG uptake rate (p>0.05). Similarly, there was no statistically significant difference between the distant lymph node metastasis positive or negative patients according to the primary tumor ^18^F-FDG uptake rate (p>0.05) (Table 2). The SUV_max_ of distant lymph node metastatic lesions was 11.0±7.0 and there was no statistically significant difference detected between TAC (11.7±5.5) and SRCC groups (9.3±9.3) (p=0.264).

## Discussion

GC still has one of the highest mortality rates among all malignancies worldwide, although 5-year survival rates have markedly increased with currently available treatments (13). The GC typically emerges between the 6^th^ and 7^th^ decade of life. National Cancer Institute (NCI) documented a median age of 69 years at diagnosis and majority of cases (81.5%) were diagnosed at ages between 55 and 84 years (14). Liu et al. (15) reported that the mean age was 58 years and that 69.8% of the patients were male and that 30.2% were female. Of 75.1% our study group was consisted of males and 24.9% females and the mean age of patients was 62.2 years. The prevalence was highest in the patients’ seventh decade of life (37.2%), followed by the sixth decade (25.9%) in this study. Advanced age and increased tumor size were described as independent prognostic risk factors in numerous published data (15,16). In a study conducted by Liu et al. (15), multivariate analysis demonstrated that age and tumor size were independent prognostic factors in both patients with SRCC and with non (N)-SRCC and also documented that the 5-year survival rates of SRCC and NSRCC group were significantly lower in patients ≥60 years old and in patients with increased size of tumor diameter. Chen et al. (16) found the mean SUV_max_ for the primary tumors significantly higher in patients ≥60 years old and increased tumor sizes. In our study, the mean SUV_max_ measured in group aged 60 years or over was found to be statistically higher than in the group aged lower than 60 years in patients with TAC.

The affinity of the primary lesion to ^18^F-FDG may be low in some types of GC and PET/CT may be false negative due to low metabolic activity especially in early-stage tumors and SRCC. Wu et al. (5) demonstrated increased ^18^F-FDG uptake as an important prognostic factor in primary lesions of GC. Similarly, Kaneko et al. (10) noted that ^18^F-FDG PET/CT scoring system may contribute in the selection of the most effective treatment modality for patients with GC and they showed some significant predictors of ^18^F-FDG uptake in primary tumor such as large tumor size, NSRCC type, and GLUT 1 expression. Chen et al. (16) showed significantly higher SUV_max_ in TAC than SRCC. In accordance with all mentioned data, the lowest SUV_max_ was detected in patients with SRCC and the primary SUV_max_ was found statistically higher in AC than SRCC in our study. In our study, there was statistically significant difference between all histological types based on ^18^F-FDG uptake. The highest SUV_max_ was obtained from medullary carcinoma and TAC groups in our study. On the other hand, Stahl et al. (17) showed that ^18^F-FDG uptake was not predictive of survival in GC.

There are some studies in the literature that investigate the relationship between primary tumor ^18^F-FDG uptake and differentiation grade in GC. Chen et al. (16) reported a higher SUV_max_ in poorly differentiated TAC than well or moderately differentiated TAC (9.579±6.474 vs. 5.452±3.722; p=0.014) in retrospective analysis of 64 patients with GC who had undergone ^18^F-FDG PET/CT. However, Yun (18) reported significantly higher mean SUV_max_ in well differentiated TAC (10.4±7.3) and moderately differentiated TAC (9.2±6.7) than in SRCC (4.4±1.8) in their study which included 126 patients with GC. In our study, there was no statistically significant difference in terms of differentiation grade in patients with TAC and SRCC.

It is well known that presence of lymph node metastases is one of the most important prognostic factors in GC (19). According to the NCI statistics, the 5-year survival rates are significantly poor for patients diagnosed as having lymph node disease (29.9%) and metastatic disease (4.5%), particularly at advanced stages (14). ^18^F-FDG PET/CT is documented to have a prominent role for detection of unsuspected metastases and nodal involvement at staging (16,18). Mukai et al. (19) detected a significantly higher rates of nodal involvement (p=0.0035) in 62 patients with GC with ^18^F-FDG PET. In a meta-analysis, the sensitivity and specificity of ^18^F-FDG PET in lymph node involvement were reported between 85.7% to 97.0%, respectively (20). In our study, when RLN detection was taken into consideration in postoperative histopathological results of 102 patients; the sensitivity, specificity, PPV and NPV for PET/CT were found 78.2%, 58.3%, 89.5% and 45.2%, respectively. According to the results of previous studies, these rates were relatively low. Although PET/CT has low sensitivity for RLN involvement, Song et al. (21) reported that preoperative lymph node ^18^F-FDG uptake in GC was an independent prognostic factor for progression and overall survival. Similarly, in a study by Kwon et al. (22) it was demonstrated that FDG uptake of lymph nodes was an independent factor contributing to recurrence free survival after curative resection in patients with advanced GC. Oh et al. (23) demonstrated that lymph node metastasis was significantly associated with primary tumor SUV_max_ (p <0.001). They described primary tumor SUV_max_ as an independent indicator of lymph node metastasis and also noted that they could not find any association between SUV_max_ and tumor location (23). Primary SUV_max_ was found statistically higher in patients with positive RLN than patients with negative RLN in our TAC and SRCC groups. Moreover, the primary tumor SUV_max_ was found to be higher in the distant metastasis positive patients than the distant metastasis negative patients in SRCC group. This finding indicated that high FDG uptake could be a poor prognostic factor in the SRCC group. There were also no statistically significant differences according to the different anatomic locations of the lesions of stomach. Smyth et al. (24) reported that ^18^F-FDG PET/CT could only able to detect the distant unsuspected metastases in approximately 10% of patients with TAC. Also, ^18^F-FDG PET/CT provided better diagnostic accuracy for the detection of lymph node and distant metastasis in patients with advanced GC (25,26).

## Conclusion

In conclusion, metabolic differences among subtypes of GC were revealed with the results of this study. Increased primary tumor SUV_max_ was associated with some clinical variables such as age and RLN metastasis in TAC. Unexpectedly, no relationship was found between distant metastatic state and primary tumor SUV_max_ in AC. However, higher SUV_max_ of primary tumor in SRCC was associated with regional, distant nodal and distant organ metastasis. Although ^18^F-FDG uptake in SRCC was lower than TAC, we think that SUV_max_ of primary tumor may be a prognostic value for this subgroup. Unfortunately, satisfactory results could not be obtained with PET/CT in regional nodal staging in this study. However, increased ^18^F-FDG uptake in RLNs could be a reliable guide to detect nodal metastasis before surgery.

## Figures and Tables

**Table 1:**
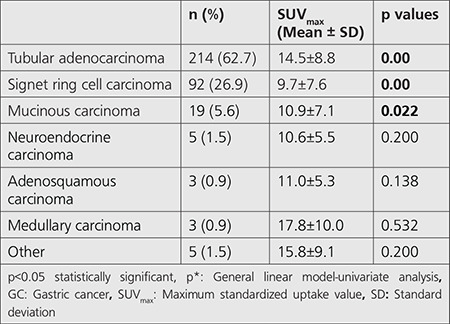
Incidences and comparison of SUV_max_ according to histopathological subtypes of GC

**Table 2:**
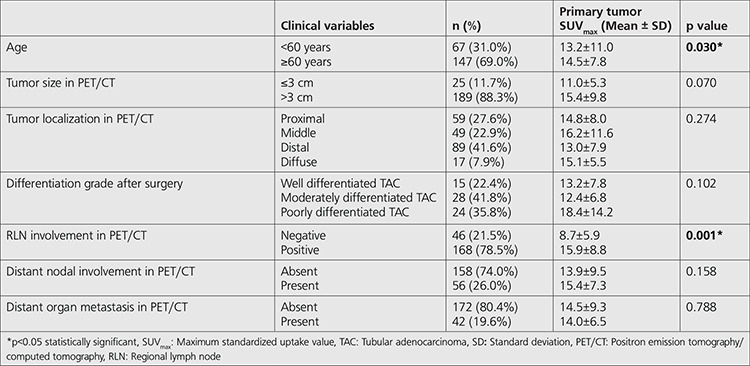
Relation between primary tumor SUV_max_ and clinical and histopathological features of the TAC-patients

**Table 3:**
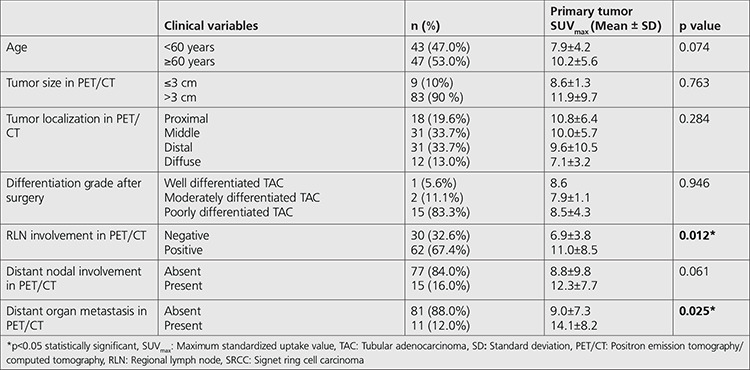
Relation between primary tumor SUV_max_ and clinical and histopathological features of the SRCC-patients

**Table 4:**
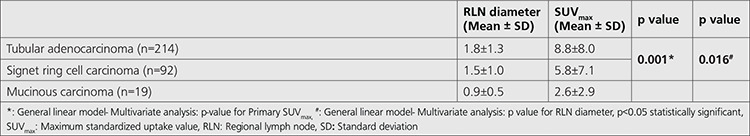
Comparison of RLN diameter and SUV_max_ in histopathological subtypes

## References

[1] Luo G, Zhang Y, Guo P, Wang L, Huang Y, Li K (2017). Global patterns and trends in stomach cancer incidence: Age, period and birth cohort analysis. Int J Cancer.

[2] Schumacher SE, Shim BY, Corso G, Ryu MH, Kang YK, Roviello F, Saksena G, Peng S, Shivdasani RA, Bass AJ, Beroukhim R (2017). Somatic copy number alterations in gastric adenocarcinomas among Asian and Western patients. PLoS One.

[3] Soares FA, Coimbra FJF, Pelosof AG, Freitas HC, Begnami MD, Costa WL, Fannelli MF, Mello CAL, Amorim MG, Pizzi MP, Caramelo L, Ferreira EN, Barros BDF, Torrezan GT, Ramalho R, Carraro DM, Chulam T, Carvalho FS, Carvalho DD, Krepischi ACV, Santos ET, Coelho LGV, Sant’Ana RO, Burbano RR, Assumpção P, Setúbal JC, Thomas AM, Chinen LTD, Braun AC, Alves V, Cassinela EK, Oliveira GP, Landemberger MC, Valieris R, Drummond R, Silva IG, Cézar R, Calsavara VF, Nóbrega CR, Bobrovnitchaia IG, Bartelli TF, Baladão GPB, Pereira ACC, Gatti CM, Abrantes LLS, Martins VR, Nunes DN, Curado MP, Neto ED;, GE4GAC group (2017). Genomics and epidemiology for gastric adenocarcinomas. Applied Cancer Research,.

[4] Shah MA, Strong VE, Boughey JC (2017). A new approach for advanced gastric cancer: Using PET scans as a biomarker of preoperative chemotherapy efficacy. Bull Am Coll Surg.

[5] Wu Z, Zhao J, Gao P, Song Y, Sun J, Chen X, Ma B, Wang Z (2017). Prognostic value of pretreatment standardized uptake value of F-18- fluorodeoxyglucose PET in patients with gastric cancer: a meta-analysis. BMC Cancer.

[6] Groheux D, Cochet A, Humbert O, Alberini JL, Hindié E, Mankoff D (2016). 18F-FDG PET/CT for staging and restaging of breast cancer. J Nucl Med.

[7] Cerfolio RJ, Bryant AS (2006). Maximum standardized uptake values on positron emission tomography of esophageal cancer predicts stage, tumor biology, and survival. Ann Thorac Surg.

[8] Lopez Guerra JL, Gladish G, Komaki R, Gomez D, Zhuang Y, Liao Z (2012). Large decreases in standardized uptake values after definitive radiation are associated with better survival of patients with locally advanced non-small cell lung cancer. J Nucl Med.

[9] Filik M, Kir KM, Aksel B, Soyda Ç, Özkan E, Küçük ÖN, İbiş E, Akgül H (2015). The role of 18F-FDG PET/CT in the primary staging of gastric cancer. Mol Imaging Radionucl Ther.

[10] Kaneko Y, Murray WK, Link E, Hicks RJ, Duong C (2015). Improving patient selection for 18F-FDG PET scanning in the staging of gastric cancer. J Nucl Med.

[11] Amin MB, Edge SB, Greene FL, Brierley JD (2017.). AJCC cancer staging manual. 8th ed. New York: Springer.

[12] Bosman FT, Carreiro F, Ralph H (2010.). Hruban, Teise N, eds. World Health Organization Classification of Tumours of the Digestive System. 4th ed. Geneva, Switzerland: WHO Press.

[13] Lee JW, Lee SM, Lee MS, Shin HC (2012). Role of 18 F-FDG PET/CT in the prediction of gastric cancer recurrence after curative surgical resection. Eur J Nucl Med Mol Imaging.

[14] SEER Cancer Statistics Factsheets: Stomach Cancer. National Cancer Institute. Bethesda, MD, Accessed in 21 Apr 2016. Available from:.

[15] Liu X, Cai H, Sheng W, Yu L, Long Z, Shi Y, Wang Y (2015). Clinicopathological characteristics and survival outcomes of primary signet ring cell carcinoma in the stomach: retrospective analysis of single center database. PLoS One.

[16] Chen R, Zhou X, Liu J, Huang G (2016). Relationship between 18F-FDG PET/CT findings and HER2 expression in gastric cancer. J Nucl Med.

[17] Stahl A, Ott K, Weber WA, Becker K, Link T, Siewert JR, Schwaiger M, Fink U (2003). FDG PET imaging of locally advanced gastric carcinomas: correlation with endoscopic and histopathological findings. Eur J Nucl Med Mol Imaging.

[18] Yun M (2014). Imaging of Gastric Cancer Metabolism Using 18 F-FDG PET/CT. J Gastric Cancer.

[19] Mukai K, Ishida Y, Okajima K, Isozaki H, Morimoto T, Nishiyama S (2006). Usefulness of preoperative FDG-PET for detection of gastric cancer. Gastric Cancer.

[20] Kim SK, Kang KW, Lee JS, Kim HK, Chang HJ, Choi JY, Lee JH, Ryu KW, Kim YW, Bae JM (2006). Assessment of lymph node metastases using 18F-FDG PET in patients with advanced gastric cancer. Eur J Nucl Med Mol Imaging.

[21] Song BI, Kim HW, Won KS, Ryu SW, Sohn SS, Kang YN (2015). Preoperative standardized uptake value of metastatic lymph nodes measured by 18F-FDG PET/CT improves the prediction of prognosis in gastric cancer. Medicine (Baltimore).

[22] Kwon HW, An L, Kwon HR, Park S, Kim S (2018). Preoperative Nodal (18)F-FDG Avidity Rather than Primary Tumor Avidity Determines the Prognosis of Patients with Advanced Gastric Cancer. J Gastric Cancer.

[23] Oh HH, Lee SE, Choi IS, Choi WJ, Yoon DS, Min HS, Ra YM, Moon JI, Kang YH (2011). The peak standardized uptake value (P-SUV) by preoperative positron emission tomography‐computed tomography (PET-CT) is a useful indicator of lymph node metastasis in gastric cancer. J Surg Oncol.

[24] Smyth E, Schöder H, Strong VE, Capanu M, Kelsen DP, Coit DG, Shah MA (2012). A prospective evaluation of the utility of 2-de- oxy-2-[(18) F]fluoro-D-glucose positron emission tomography and computed tomography in staging locally advanced gastric cancer. Cancer.

[25] Kawanaka Y, Kitajima K, Fukushima K, Mouri M, Doi H, Oshima T, Niwa H, Kaibe N, Sasako M, Tomita T, Miwa H, Hirota S (2016). Added value of pretreatment (18)F-FDG PET/CT for staging of advanced gastric cancer: comparison with contrast-enhanced MDCT. Eur J Radiol.

[26] Altini C, Niccoli Asabella A, Di Palo A, Fanelli M, Ferrari C, Moschetta M, Rubini G (2015). 18F-FDG PET/CT role in staging of gastric carcinomas: comparison with conventional contrast enhancement computed tomography. Medicine (Baltimore).

